# Activation of high and low affinity dopamine receptors generates a closed loop that maintains a conductance ratio and its activity correlate

**DOI:** 10.3389/fncir.2013.00169

**Published:** 2013-10-22

**Authors:** Wulf-Dieter C. Krenz, Ryan M. Hooper, Anna R. Parker, Astrid A. Prinz, Deborah J. Baro

**Affiliations:** ^1^Department of Biology, Georgia State UniversityAtlanta, GA, USA; ^2^Department of Biology, Emory UniversityAtlanta, GA, USA; ^3^Department of Biomedical Engineering, Georgia Institute of Technology and Emory UniversityAtlanta, GA, USA

**Keywords:** activity-dependent intrinsic plasticity, metaplasticity, metamodulation, HCN channel, stomatogastric, pyloric network

## Abstract

Neuromodulators alter network output and have the potential to destabilize a circuit. The mechanisms maintaining stability in the face of neuromodulation are not well described. Using the pyloric network in the crustacean stomatogastric nervous system, we show that dopamine (DA) does not simply alter circuit output, but activates a closed loop in which DA-induced alterations in circuit output consequently drive a change in an ionic conductance to preserve a conductance ratio and its activity correlate. DA acted at low affinity type 1 receptors (D1Rs) to induce an immediate modulatory decrease in the transient potassium current (*I*_A_) of a pyloric neuron. This, in turn, advanced the activity phase of that component neuron, which disrupted its network function and thereby destabilized the circuit. DA simultaneously acted at high affinity D1Rs on the same neuron to confer activity-dependence upon the hyperpolarization activated current (*I*_h_) such that the DA-induced changes in activity subsequently reduced *I*_h_. This DA-enabled, activity-dependent, intrinsic plasticity exactly compensated for the modulatory decrease in *I*_A_ to restore the *I*_A_:*I*_h_ ratio and neuronal activity phase, thereby closing an open loop created by the modulator. Activation of closed loops to preserve conductance ratios may represent a fundamental operating principle neuromodulatory systems use to ensure stability in their target networks.

## INTRODUCTION

Neuromodulators reconfigure circuit output; but, they must confer stability as well as flexibility in order to maintain the functionality of a target network. Our knowledge of modulatory stabilizing mechanisms is limited. We suggest that modulators stabilize circuits by activating feedback loops that preserve conductance ratios and their activity correlates. Many cells maintain conductance ratios (Linsdell and Moody, 1994; MacLean et al., 2003; Schulz et al., 2006; Peng and Wu, 2007), and it is generally thought that a given conductance ratio sustains a specific activity parameter(s) (Marder and Goaillard, 2006; Hudson and Prinz, 2010; Soofi et al., 2012). A neuromodulator could establish a feedback loop if it modulated one of the conductances in the pair and conferred activity dependence on the other. In this case, modulation of the first current would contribute to changes in neuronal and circuit output that, in turn, would drive a change in the second current to restore the ratio and the activity feature. The work presented here establishes, for the first time, the existence of such a feedback loop.

**FIGURE 1:**
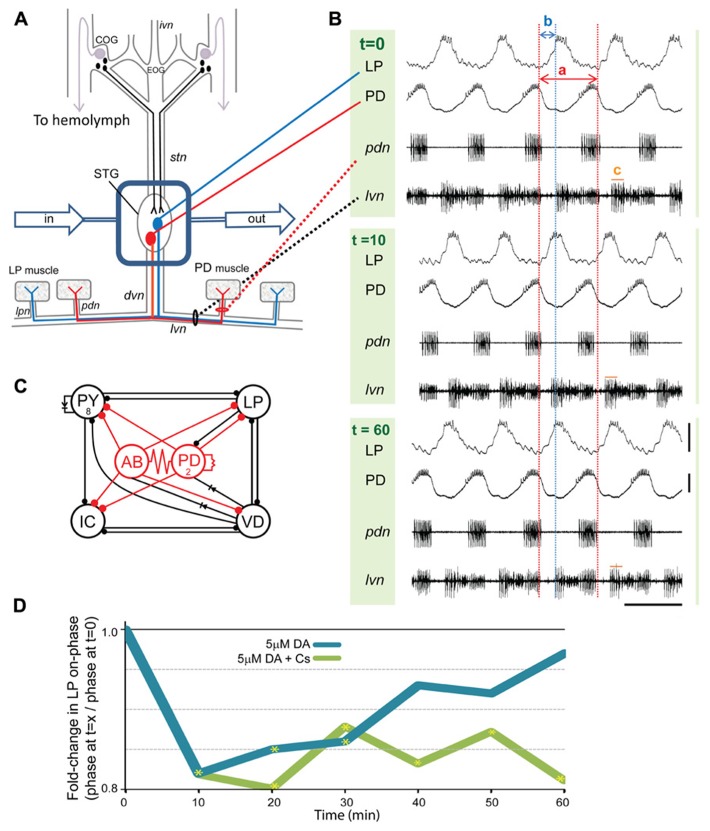
**Phase recovery in the pyloric network.** **(A)** *In situ* preparation: the stomatogastric nervous system (STNS) is dissected and pinned in a dish. The commissural ganglia (CoGs) contain DA neurons that project to the STG (black) and L-cells, which are the source of neurohormonal DA (purple). The well surrounding the STG (blue rectangle) is continuously superfused with saline (in/out arrows). There are ~30 neurons in the STG. The pyloric network comprises 14 STG neurons; two are drawn: pyloric dilator (PD, red), lateral pyloric (LP, blue). Network neurons interact locally within the STG and can project axons to striated muscles surrounding the foregut. The diagram shows that PD and LP neurons project their axons through identified nerves to innervate muscles (rectangles). **(B)** Spontaneous pyloric network output from one experiment during a 1 h 5 μM DA application: one set of traces comprises two intra-cellular recordings (top) and two extra-cellular recordings (bottom) from the *in* *situ* preparation diagrammed in **(A)**. The three sets of traces represent recordings from the indicated time points, in minutes, directly before and after the start of DA application. Red and blue dashed lines reveal how cycle period and LP-on delay change with time. The two red lines demarcate one cycle. Cycle period (a) is defined as the time between the last spike in one PD burst and the last spike in the subsequent PD burst. Note that for each time point the last spike in the first PD burst is aligned with the first red line; however, the last spike in the second PD burst is not aligned with the second red line except at *t* = 0. This is because 5 μM DA produces a sustained average 10% reduction in cycle period. Thus, for *t* = 10 and 60 min, the spike in the second PD burst occurs prior to the second red line. Within the indicated cycle, a blue line aligns with the first spike in LP at *t* = 0. The time between the last spike in PD and the first spike in LP (b) represents LP-on delay, and LP-on phase is: b/a. Note that for the *t* = 10 min cycle, the first spike in LP occurs well before the blue line. This is because DA produces an average~20% LP-on phase advance. LP-on phase recovery can be seen in the cycle at *t* = 60 min because the first LP spike is again aligned with the blue line. Measures of pyloric output parameters can be obtained from either intra- or extra-cellular traces, and LP burst duration is indicated by (c) on the extracellular traces; scale bars: 20 mV and 500 ms. **(C)** The pyloric circuit: the diagram represents pyloric neuron interactions within the STG. Open circles represent the six cell types, numbers indicate more than one cell within a cell type: anterior burster (AB), inferior cardiac (IC), ventricular dilator (VD); filled circles, inhibitory chemical synapses; resistors and diodes, electrical coupling; red, pacemaker kernel and its output connections. **(D)** Phase recovery: the preparation shown in **(A)** was superfused with one of the two indicated treatments for 1 h and LP on-phase was measured every 10 min throughout the experiment (*n* ≥ 6/treatment). Average fold-changes in LP on-phase are plotted for each group; yellow asterisks, significantly different from *t* = 0, data taken from Rodgers et al. (2011a). Note that phase recovery in 5 μM DA was blocked by Cs.

The 14-neuron pyloric circuit in the spiny lobster, *Panulirus interruptus*, is a small central pattern generator (CPG) that drives the striated muscles surrounding the pylorus to produce an ordered series of contractions (Marder and Bucher, 2007). One cycle of contractions is continuously repeated to produce constant filtering of the foregut contents. The repetitive cycle of muscle contractions is underpinned by the recurrent output of the pyloric CPG, which stems from a pacemaker kernel that rhythmically inhibits four follower neuron cell types. The follower neurons then display post-inhibitory rebound (PIR), and differences in their rates of PIR, together with the synaptic architecture, produce a tri-phasic motor pattern (**Figure 1**).

Follower neuron cell types have specific activity phases, meaning that a given cell type fires a burst of action potentials at the same point in each reiteration of the cyclic network output. The timing of neuronal activity phases is determined, in part, by their rate of PIR. *I*_A_ and *I*_h_ are opposing subthreshold conductances whose ratio regulates the rate of PIR (Harris-Warrick et al., 1995). Population studies on other species of crustaceans showed that pyloric neuron activity phases (Bucher et al., 2005; Goaillard et al., 2009) and their *I*_A_:*I*_h_ ratios (Temporal et al., 2012) were invariant across individuals and lifetimes, suggesting compensatory mechanisms may exist to maintain the *I*_A_:*I*_h_ ratio and its activity correlates. Such a compensatory mechanism(s) was revealed by overexpressing the Kv4 channels mediating *I*_A_ throughout days in organ culture. Overexpression of *I*_A_ in pyloric neurons resulted in compensatory increases in *I*_h_ that maintained the rates of PIR (MacLean et al., 2003, 2005).

We suggest that a mechanism to maintain the *I*_A_:*I*_h_ ratio may also prevail during DA modulation of pyloric neurons (Rodgers et al., 2011a): there is a single LP follower neuron in the pyloric network, and it contributes to cycle frequency regulation (Weaver and Hooper, 2003). The timing of the LP activity phase is critical for this function (Johnson et al., 2011). LP expresses D1Rs but not D2Rs (Zhang et al., 2010), and a 10 min bath application of 5 μM DA can disrupt the LP *I*_A_:*I*_h_ ratio and induce an LP phase advance largely by decreasing LP *I*_A_ (Harris-Warrick et al., 1995). DA modulation also decreases LP burst duration and increases pyloric cycle frequency through intrinsic and network effects (Harris-Warrick et al., 1998; Rodgers et al., 2011a). During continuous DA application, the timing of LP activity phase is restored, while the DA-induced changes in burst duration and cycle frequency are maintained (Rodgers et al., 2011a); thus, a compensatory mechanism operates to restore neuronal activity phase during neuromodulation. Here we investigate this mechanism and show that LP phase recovery involves a DA- and activity-dependent (DAD) decrease in LP *I*_h_ that compensates for the modulatory decrease in LP *I*_A_ to restore the LP *I*_A_:*I*_h_ ratio and LP activity phase.

## MATERIALS AND METHODS

### ANIMALS AND DRUGS

California spiny lobsters, *Panulirus interruptus*, were purchased from Catalina Offshore Products (San Diego, CA, USA) and Marinus Scientific (Long Beach, CA, USA) and housed at 16–18°C in saltwater aquaria at Georgia State University (Atlanta, GA, USA). Animals of both sexes were used in these experiments. TTX was from Tocris (Ellisville, MO, USA), all other reagents were from Sigma (St. Louis, MO, USA). Solutions containing DA were made fresh every 30 min in saline to prevent oxidation and reduced DA activity.

### PHYSIOLOGICAL RECORDINGS

Lobsters were anesthetized on ice for at least 30 min, followed by dissection of the stomatogastric nervous system (**Figure 1**), as previously described (Panchin et al., 1993). A Vaseline well was constructed around the stomatogastric ganglion (STG) which was continuously superfused for the remainder of the experiment with *Panulirus* (*P*.) saline (in mM: 479 NaCl, 12.8 KCl, 13.7 CaCl_2_, 39 Na_2_SO_4_, 10 MgSO_4_, 2 glucose, 4.99 HEPES, 5 TES; pH 7.4). Experiments were conducted at room temperature (19–21°C). Temperature was continuously monitored with a miniature probe inside the well. Temperatures changed by less than 1°C throughout the course of the day.

Cells were identified by combining standard intracellular and extracellular recording techniques. Lateral pyloric (LP) neurons were identified by their distinct waveforms, the timing of their voltage oscillations, and correlation of spikes on the extracellular and intracellular recordings. Intracellular somatic LP recordings were obtained using 20–40 MΩ glass microelectrodes filled with 3 M KCl connected to Axoclamp 2B or 900A amplifiers (Molecular Devices, Foster City, CA, USA). Extracellular recordings of identified motor neurons were obtained using a model 1700 differential AC amplifier (A-M Systems, Everett, WA, USA) and stainless steel pin electrodes on the lateral ventricular nerve *(lvn)* and pyloric dilator nerve *(pdn)* and recorded with Axoscope v8.2 software (Molecular Devices, Foster City, CA, USA). Extracellular recordings were analyzed using DataView v6.3.2 (Heitler, 2009) to determine cycle period, spike frequency, burst duration, LP-on/off delays, and LP activity phase as previously described (Rodgers et al., 2011a). Reported values for all parameters represent a 10 cycle average.

Experiments in TTX blocked action potential firing and slow voltage oscillations in STG neurons. Under these conditions, the resting membrane potential of most pyloric neurons is between ~ -52 and -62 mV. Pyloric neuron input/output curves suggest that graded transmitter release will be minimal to non-existent at these voltages (Johnson et al., 1991, 1995). DA (100 μM) can shift the curves (Johnson and Harris-Warrick, 1990), but a 10-fold lower concentration has a minimal effect on the strength of graded release (Kvarta et al., 2012). Pyloric neurons can oscillate in TTX if bathed in 100 μM DA, but we do not observe pyloric oscillations in TTX at ≤5 μM DA.

### TWO-ELECTRODE VOLTAGE CLAMP (TEVC)

**FIGURE 2:**
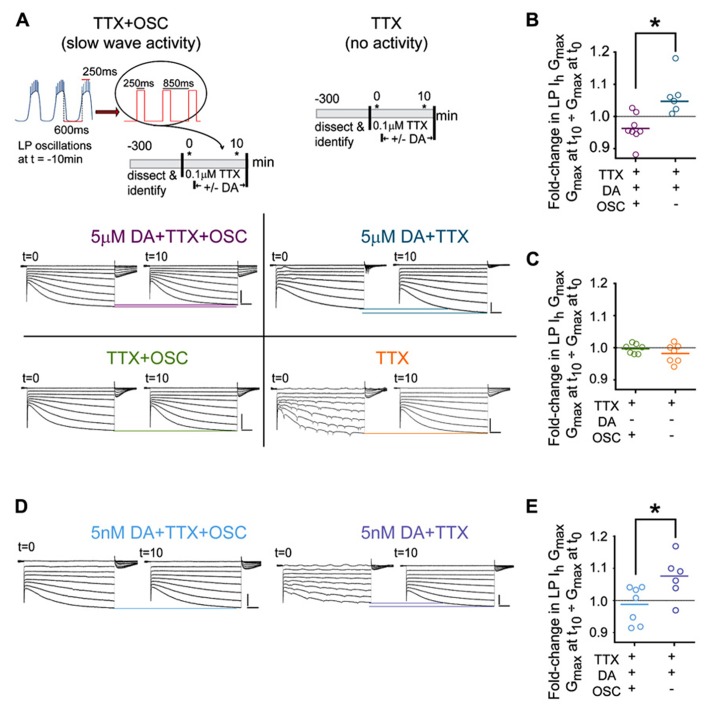
**DA-enables activity-dependent alterations in LP *I*_h_. (A)** The protocols used to measure DA- and/or activity-induced changes in LP *I*_h_ are diagramed in the top two panels. Asterisks indicate points where TEVC measures of LP *I*_h_ were made. Bottom panels show typical LP *I*_h_ recordings at *t* = 0 and *t* = 10 min for each of the four the indicated treatment groups; scale bars: 500 ms and 5 nA. Note that distal compartments of LP neurons are not completely space clamped and oscillatory activity at *t* = 0 was observed in all treatment groups in ~20% of the experiments due to the short exposure to TTX (example seen in TTX group); nevertheless, *I*_h_ could be measured from the traces. **(B,C)** Plots of the fold-changes in LP *I*_h_ *G*_max_ in each treatment group at *t* = 10 min. Each symbol represents one experiment; solid lines indicate the means; *p < 0.05, *t*-tests. **(D)** Typical LP *I*_h_ recordings for additional experiments in 5 nM DA. **(E)** Plots of the fold-changes in LP *I*_h_ *G*_max_ in each treatment group in 5 nM DA at *t* = 10 min. Each symbol represents one experiment; solid lines represent means *p < 0.05, *t*-test.

For TEVC of LP *I*_h_, the LP neuron was impaled with two micropipettes (8–10 MΩ when filled with 3 M KCl) connected to Axoclamp 2B or 900A amplifiers (Molecular Devices, Foster City, CA, USA). The well surrounding the STG was superfused with P. saline containing 100 nM TTX for ≥5 min. LP was clamped to a -50 mV holding potential using pClamp software. *I*_h_ was elicited using a series of 4 s hyperpolarizing voltage steps, from -60 to -120 mV in 10 mV increments with 6 s between steps. Steady-state peak currents were measured by fitting the current trace back to the beginning of the hyperpolarizing voltage step using a single exponential equation. In some experiments small oscillations interrupted the current trace at *t* = 0 (e.g., **Figure 2**) and prevented curve fitting. In those experiments, peak *I*_h_ was measured by subtracting the initial fast leak current from the slowly developing peak of *I*_h_ at the end of each negative voltage step. Currents were converted to conductance using (*G* = *I*_peak_/(*V*_m_ - *V*_rev_) and fitted to a first-order Boltzmann equation. *V*_rev_ *I*_h_ = -35 mV (Kiehn and Harris-Warrick, 1992). For TEVC measurement of peak *I*_A_ the command potential was stepped from -50 to -90 mV for 200 ms to remove resting inactivation. The deinactivating prepulse was immediately followed by an activation pulse to 60 mV for 400 ms to ensure that channels were maximally activated and observed changes could not be due to alterations in voltage dependence (Zhang et al., 2010). To subtract the leak current the hyperpolarizing prepulse was omitted and instead the prepulse was set to -40 mV to remove *I*_A_ activation from the -50 mV holding potential. For recordings to measure the LP *I*_A_:*I*_h_ ratio in 5 μM DA, the saline also contained 20 μM TEA and 1 μM PTX to block DA-induced modulatory changes in other conductances that could interfere with measures of peak currents. Recurring voltage steps to mimic slow wave oscillations and action potentials were constructed with pClamp software. When currents were not being measured, and recurring steps were not being implemented, LP was held at its initial resting membrane potential in TTX (on average, -59 mV).

### DYNAMIC CLAMP

**FIGURE 3:**
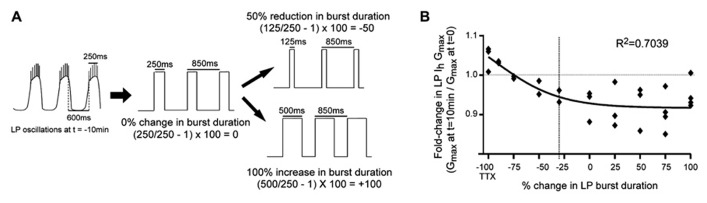
**LP *I*_h_ activity-dependence curve in 5 μM DA. (A)** Experimental protocol: TEVC was used to create a recurring voltage step that mimicked slow wave activity at *t* = -10 min, except the length of the depolarizing step varied across experiments to alter burst duration. Examples are shown for how the length of the step corresponded to no change, a reduction or an increase in burst duration. There was no change in cycle period. **(B)** Plot of fold-changes in LP *I*_h_ *G*_max_ for the 10 min time point; -100 on the *x*-axis represents experiments in TTX without a recurring step; vertical dashed line marks 30% reduction in burst duration (i.e., average 5 μM DA-induced change) each diamond represents one experiment; data were fitted with a Boltzmann sigmoidal equation.

We used the dynamic clamp to introduce an artificial injection current (*I*_inj_) specified to counteract the metaplastic (DA modulation of activity dependent (AD) intrinsic plasticity) change in *I*_h_ in LP neurons during ongoing rhythmic pyloric activity following bath application of 5 μM DA (Sharp et al., 1993a,b; Prinz et al., 2004a). The membrane potential of the LP soma was amplified, fed into a PCI-6052E DAQ board (National Instruments, Austin, TX, USA), and digitized at 20 kHz. The dynamic clamp program was written in the C programming language and designed to use the real time Linux dynamic controller (Dorval et al., 2001). This dynamic clamp software calculated the *I*_inj_ that would be active at the measured membrane potential (*V*_m_) given a set of model parameters as follows:

$$I_{inj}=G_{\max}m(V_{m}-E_{rev}),$$

where *m* changed according to *dm/dt* = (*m*_∞_ - m)/τ _m_, computed numerically using the first-order forward Euler method, and *m*_∞_ was given by *m*_∞_ = 1/(1 + exp((*V*_m_ - *V*_½_)/*V*_slope_). *E*_rev_ was set to -35 mV (Kiehn and Harris-Warrick, 1992). Values for *I*_h_ τ_m_ represent average TEVC measures from 12 experiments. LP *I*_h_ was measured in each LP neuron before the dynamic clamp experiment and *G*_max_, *V*_½_, and *V*_slope_ were determined from a Boltzmann fit as described above. The predicted metaplastic change in LP *I*_h_ *G*_max_ was determined using the activity-dependence curve in **Figure 3** and the measured change in LP burst duration after a 10 min application of 5 μM DA. The predicted metaplastic change in *I*_h_ conductance was subtracted with the dynamic clamp, which calculated and continuously injected current according to the above model, where *G*_max_ = measured LP *I*_h_ *G*_max_ × predicted metaplastic change in LP *I*_h_ *G*_max_. Intracellular and extracellular recordings of LP activity throughout the experiment were obtained using a separate computer equipped with Axoscope and Clampex 9.2 software (Axon Instruments).

### STATISTICAL ANALYSIS

Data were checked for normal distribution and analyzed using parametric statistical tests with Prism software package v5.01 (GraphPad, La Jolla, CA, USA). Significance was set at *p* < 0.05 in all cases. Individual samples that were more than 2 standard deviations from the mean were excluded from the analyses after determining the mean. This eliminated two experiments. ANOVAs are followed by *post hoc* tests that make all possible comparisons between columns (Tukey’s) or that compare all columns to a single column, usually *t* = 0 (Dunnett’s). Means are followed by standard errors.

## RESULTS

### THE EXPERIMENTAL MODEL

The pyloric circuit is located in the crustacean STG (**Figure 1A**), and it produces a rhythmic motor output *in situ*. Each pyloric cell type displays repetitive oscillations in membrane potential with a burst of spikes riding on the depolarized plateau (**Figure 1B**). The circuit comprises six oscillatory cell types coupled by fast inhibitory synapses and/or gap junctions (**Figure 1C**). The pacemaker kernel (anterior burster (AB) + 2 PD neurons) rhythmically inhibits the four follower neuron cell types, which then display different rates of PIR. The different rates of PIR are due, in part, to differences in the expression of *I*_A_ in each follower neuron (Baro et al., 1997, 2000). *I*_A_ delays pyloric neuron PIR (Tierney and Harris-Warrick, 1992): the hyperpolarizing phase of the membrane potential oscillation removes resting inactivation from the Kv4 channels mediating *I*_A_ and activates the hyperpolarization activated cyclic nucleotide (HCN) gated channels mediating the depolarizing inward *I*_h_. The subsequent depolarization activates Kv4 channels, and the resulting outward potassium current slows the rate of depolarization. In this way, the ratio of *I*_A_:*I*_h_ can influence when LP activity phase begins (termed LP-on phase). **Figure 1B** shows intra- and extra-cellular recordings from a typical experiment where the STG was superfused with 5 μM DA for 1 h: DA was applied after the initial recording at *t* = 0. By 10 min, DA increased pyloric network cycle frequency by reducing the inherent period of the pacemaker AB neuron (Harris-Warrick et al., 1998; Rodgers et al., 2011a). DA application also reduced LP burst duration and advanced LP firing phase. The traces indicate that by 60 min in DA, network cycle frequency was still increased and LP burst duration was still decreased, but LP-on phase was restored. In previous experiments we clearly demonstrated that phase recovery was AD: if the experiment shown in **Figure 1B** was repeated with continuous injection of a depolarizing bias current into LP to block the DA-induced decrease in LP burst duration, then the LP phase advance occurred, but phase recovery did not (Rodgers et al., 2011a). We also showed that phase recovery in the presence of 5 μM DA could be blocked by bath application of CsCl to reduce *I*_h_ (**Figure 1D**).

### DA- AND ACTIVITY-DEPENDENT (DAD) REGULATION OF LP *I_h_* IN 5 μM DA

We first tested the idea that DA conferred activity-dependence upon LP *I*_h_ by measuring *I*_h_ in LP neurons that showed different activity patterns. In these experiments, LP neurons have one of two activity patterns: either LP activity is completely blocked (TTX), or LP displays normal slow wave but not spike activity (TTX + OSC). LP *I*_h_ is measured in each of these two groups in the presence and absence of DA resulting in four treatment groups. The experiment, which is diagrammed in **Figure 2A**, was as follows: after dissection and cell identification, the STG was superfused with TTX for 5 min to block spike and slow wave activity, and the TTX was present throughout the remainder of the experiment. Next, at *t* = 0, LP *I*_h_ was measured with somatic TEVC. After the first measure of LP *I*_h_, DA was or was not added to the superfusate and LP *I*_h_ was re-measured after 10 min. The voltage of LP was continuously controlled with TEVC throughout the experiment. Between measures of LP *I*_h_, a recurrent step mimicking LP slow oscillatory activity at *t* = -10 min was (TTX + OSC) or was not (TTX) implemented. Frequency, duration, and amplitude of the recurrent steps were chosen for each preparation individually depending upon measured activity at *t* = -10 min: frequency and duration of the recurrent step corresponded to average cycle frequency and LP burst duration at *t* = -10 min, respectively; the step and holding potentials corresponded to the average peak and nadir of the LP oscillation at *t* = -10 min, respectively. In the absence of the recurring voltage step, LP was held at its initial resting membrane potential in TTX (-59 mV on average). Typical LP *I*_h_ recordings for each treatment group are shown in **Figure 2A**.

The results indicated that DA conferred activity dependence upon LP *I*_h_: in the presence of DA, the fold-change in LP *I*_h_ *G*_max_ varied according to LP activity (**Figure 2B**; *t*-test, *p* < 0.004); by 10 min in 5 μM DA average LP *I*_h_ *G*_max_ was significantly decreased in preparations with the slow wave LP activity pattern (paired *t*-test, *t* = 0 vs. 10 min, *p* = 0.0491) and significantly increased in preparations showing no LP activity (paired *t*-test, *p* = 0.0285). In the absence of DA the fold-change in LP *I*_h_ *G*_max_ was not significantly different between treatment groups (**Figure 2C**, *t*-test, *p* = 0.256) and there was no significant change in LP *I*_h_ *G*_max_ by *t* = 10 min relative to *t* = 0 in preparations where slow wave activity was mimicked (paired *t*-test, *p* = 0.1166) or activity was completely blocked (Wilcoxon matched pairs signed rank test, *p* = 0.2969). We previously demonstrated that 5 nM DA acting at high affinity LP D1Rs permitted a decrease in LP burst duration to produce an increase in LP *I*_h_ *G*_max_ that persisted well beyond DA washout (Rodgers et al., 2011a). This suggested that perhaps high affinity D1Rs receptors might also mediate the more rapid DAD regulation of LP *I*_h_ *G*_max_ observed in **Figure 2B**. To test this hypothesis, we repeated the experiments diagrammed in **Figure 2A**, but applied 5 nM rather than 5 μM DA (**Figure 2D**). The results were consistent with the hypothesis; in the presence of 5 nM DA, the fold-change in LP *I*_h_ *G*_max_ at *t* = 10 min varied according to activity (**Figure 2E**, *t*-test, *p* = 0.0321). Interestingly, LP *I*_h_ *G*_max_ did not change over time in 5 nM DA preparations where slow wave activity was mimicked (paired *t*-test, *t* = 0 vs. 10 min, *p* = 0.5962); however, a complete block of activity produced a clear trend toward an increase in LP *I*_h_ *G*_max_ (paired *t*-test, *p* = 0.0596), and the magnitude of the increase was similar to that observed in 5 μM DA (compare Figures 2B vs. 2E). The difference in the TTX + OSC treatment groups in 5 nM DA (no change in *G*_max_) vs. 5 μM DA (decrease in *G*_max_) may be due to the fact that micromolar DA can regulate calcium dynamics during oscillations in membrane potential (Johnson et al., 2003; Kadiri et al., 2011). For all treatment groups the voltages of half activation changed by ≤2.3 mV on average, and LP *I*_h_ voltage dependence is not considered further here. In sum, ≥5 nM DA permitted activity to differentially regulate LP *I*_h_ *G*_max_; but, neither 5 nM DA alone nor changes in activity alone significantly altered LP *I*_h_ *G*_max_; i.e., DA did not modulate LP *I*_h_, but conferred activity-dependence upon LP *I*_h_.

### DAD REGULATION OF LP *I_h_* IS NECESSARY FOR PHASE RECOVERY

Our previous study suggested that LP phase recovery during sustained DA modulation was triggered by a change in LP burst duration (Rodgers et al., 2011a). In order to understand if and how DAD regulation of LP *I*_h_ restored the timing of the LP activity phase in 5 μM DA, it was necessary to determine how LP *I*_h_ varied according to changes in LP burst duration. An LP *I*_h_ activity-dependence curve for changes in burst duration was constructed by repeating the previous experiments in 5 μM DA for the TTX + OSC treatment group, except that the length of the depolarizing step varied across experiments to mimic a change in burst duration (**Figure 3A**). A plot of the fold-change in LP *I*_h_ *G*_max_ vs. percent change in LP burst duration at *t* = 10 min was best-fitted with a Boltzmann sigmoidal equation. DA (5 μM) produced an average 30% decrease in LP burst duration (Rodgers et al., 2011a), and so, according to the activity-dependence curve, LP *I*_h_ *G*_max_ should be reduced by ~6% in 5 μM DA during on-going activity (**Figure 3B**, dashed line). This decrease in LP *I*_h_ is consistent with our hypothesis that DAD regulation of LP *I*_h_ compensates for the DA-induced modulatory decrease in LP *I*_A_ to restore the *I*_A_:*I*_h_ ratio and the timing of LP activity phase.

**FIGURE 4:**
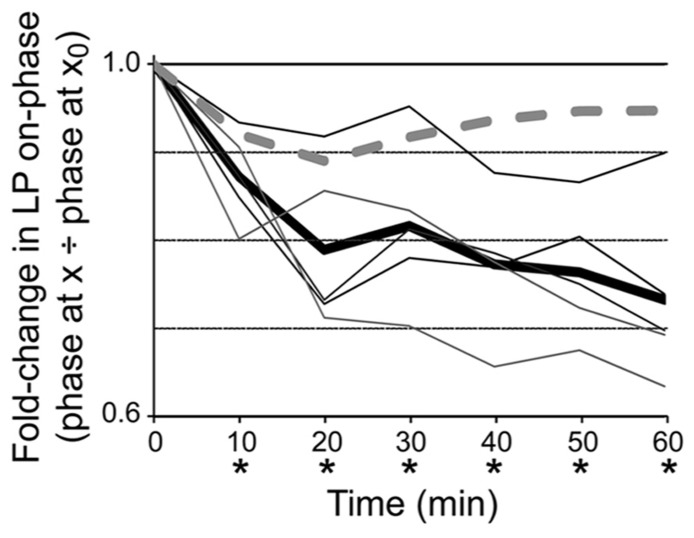
**DAD regulation of LP *I*_h_ is necessary for phase recovery in 5 μM**. Plots of fold-changes in LP-on phase over time for dynamic clamp (solid lines) and control (dashed line) experiments indicate that introduction of a dynamic clamp current to abrogate DAD regulation of LP *I*_h_ prevents phase recovery; thin lines, individual experiments with dynamic clamp (*n* = 5); thick line, average for experiments with dynamic clamp; dashed line, control experiment that was exactly the same as the dynamic clamp experiments except that the dynamic clamp was turned off during the 1 h superfusion with 5 μM DA. Repeated measures ANOVA with Dunnett’s *post hoc* tests that compared all time points to *t* = 0 showed that average LP-on phase did not recover in experimental preparations, *F*(6,4) = 16.04, *p* < 0.0001; *p < 0.05. Note that phase did recover in the control experiment.

In order to determine if DAD regulation of LP *I*_h_ was necessary for phase restoration, we used the activity-dependence curve in conjunction with dynamic clamp experiments to abrogate DAD regulation of LP *I*_h_ (**Figure 4**). The experimental preparation was as shown in **Figure 1A**. After dissection and cell identification the STG was superfused with TTX for 5 min; LP *I*_h_ was measured with TEVC and values for *G*_max_, *V*_1/2 _and V_slope_ were subsequently incorporated into the dynamic clamp model for *I*_inj_ (see Section “Materials and Methods”). TTX was immediately washed out with saline for 90 min. LP burst duration was measured at the end of the wash followed by application of 5 μM DA from *t* = 0–60 min. The predicted fold-change in LP *I*_h_ *G*_max_ due to DAD regulation was determined using the activity-dependence curve in **Figure 3** and the measured change in LP burst duration from *t* = 0 to *t* = 10 min, and was subsequently incorporated into the dynamic clamp model for *I*_inj_ (see Section “Materials and Methods”). From ~*t* = 10 to 60 min, dynamic clamp was used to remove the predicted DAD regulation of LP *I*_h_, i.e., to add back, in the form of dynamic clamp current, the same amount of *I*_h_ that was predicted to have been lost because of DAD regulation. LP-on phase was subsequently measured every 10 min from *t* = 0–60 min. Plots of the fold-change in LP-on phase over the course of the experiment demonstrated that 5 μM DA-induced the usual phase advance, but removing DAD regulation of LP *I*_h_ prevented LP-on phase recovery (compare Figures 4 vs. 1D). It also prevented LP-off phase recovery [repeated measures ANOVA: *F*(6,4) = 3.119, *p* = 0.0210]. However, it should be noted that the recovery of LP-off phase may be complicated by the PY cell activity phase. The PY-LP synapse contributes to the timing of LP-off phase, especially in DA; thus, any change in LP-on phase that subsequently alters the timing of PY activity through the LP–PY synapse may also indirectly affect LP-off phase (Johnson et al., 2011). From these experiments we conclude that DAD regulation of LP *I*_h_ *G*_max_ is necessary for LP-on phase restoration.

### DAD REGULATION OF LP *I_h_* COMPENSATES FOR MODULATORY CHANGES IN LP *I_A_* TO RESTORE *I_A_*:*I_h_*

**FIGURE 5:**
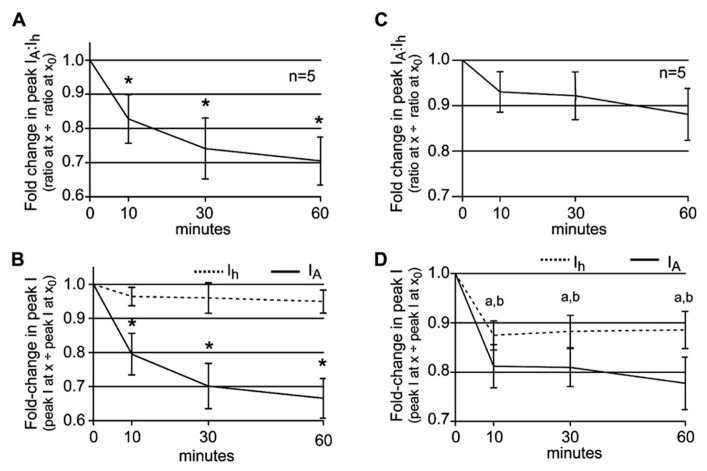
**The LP *I*_A_:*I*_h_ ratio is maintained in 5 μM DA when DA application is accompanied by DA-induced changes in slow wave activity. (A)** A plot of the fold-changes in the LP *I*_A_:*I*_h _ratio (mean ± SEM) throughout a 1 h superfusion with 5 μM DA and implementation of a recurring voltage step that mimicked the DA-induced 30% decrease in LP burst duration, but no change in cycle frequency. The ratio significantly decreased with time; repeated measures ANOVA with Dunnett’s *post hoc* tests that compare all time points to *t* = 0, *F*(3,4) = 7.322, *p* = 0.0032. **(B)** Plots of the fold-changes in peak LP *I*_A_ and *I*_h_ (mean ± SEM) from the same experiments as in **(A)**. Repeated measures ANOVAs with Dunnett’s *post hoc* tests that compare all time points to *t* = 0 indicate that only LP *I*_A_ was significantly decreased [LP *I*_A_: *F*(3,4) = 19.66, *p* < 0.0001; LP *I*_h_, *F*(3,4) = 1.218, *p* = 0.3456]. *p < 0.05. **(C)** Plot of the fold-changes in the LP *I*_A_:*I*_h _ratio (mean ± SEM) throughout a 1 h superfusion with 5 μM DA and implementation of a recurring voltage step that mimicked the DA-induced 30% decrease in LP burst duration and a 10% increase in cycle frequency. The ratio did not change significantly over time (repeated measures ANOVA, see text). **(D)** Plots of the fold-changes in peak LP *I*_A_ and *I*_h_ (mean ± SEM) from the same experiments as in **(C)** show that both currents are stably altered by 10 min; a and b indicate a significant change in LP *I*_A_ and *I*_h_, respectively, based on repeated measures ANOVA with Dunnett’s *post hoc* tests that compare all time points to *t* = 0, *p* < 0.05 (see text).

Thus far the data are consistent with our working model for how phase advance and recovery occur in 5 μM DA: 5 μM DA initially alters the LP *I*_A_:*I*_h_ ratio by decreasing LP *I*_A_, and this creates a phase advance (Harris-Warrick et al., 1995; Zhang et al., 2010). DA (5 μM) also produces a 30% reduction in LP burst duration, and this subsequently initiates a process that generates a compensatory decrease in LP *I*_h_ to restore the LP *I*_A_:*I*_h_ ratio and produce phase recovery. In order to further test this hypothesis, we repeatedly measured the LP *I*_A_:*I*_h_ ratio during a 1 h 5 μM DA application accompanied by a recurrent step that mimicked a 30% reduction in LP burst duration. At *t* = 0, peak LP *I*_A_ was measured at +60 mV and peak LP *I*_h_ was measured at -120 mV. DA (5 μM) was immediately applied for 1 h and peak currents were re-measured at *t* = 10, 30, and 60 min. During the DA application, whenever peak currents were not measured, LP received a recurring step. Plots of the average fold-changes in the peak *I*_A_:*I*_h_ ratio (**Figure 5A**) and average peak *I*_A_ and *I*_h_ (**Figure 5B**) suggested that our hypothesis was incorrect or incomplete. The average *I*_A_:*I*_h_ ratio significantly decreased over time (**Figure 5A**) because the decreases in peak LP *I*_h_ did not fully compensate for the decreases in peak LP *I*_A_ (**Figure 5B**).

It is noteworthy that DA-induced a change in both LP burst duration and cycle period (Rodgers et al., 2011a), but our step only mimicked the change in burst duration. We next asked if the DA-induced increase in cycle frequency contributed to DAD regulation of LP *I*_h_ *G*_max_, by repeating the experiments to measure the LP *I*_A_:*I*_h_ ratio but using a recurring voltage step that mimicked both the average 30% decrease in LP burst duration and the 10% increase in cycle frequency. In this case, the average *I*_A_:*I*_h_ ratio did not change significantly throughout the experiment [**Figure 5C**, repeated measures ANOVA, *F*(3,4) = 2.161, *p* = 0.1457], despite the fact that by 10 min, average peak LP *I*_A_ was significantly and stably reduced to 81 ± 4% of its initial value [**Figure 5D**, repeated measures ANOVA, *F*(3,4) = 16.91, *p* = 0.0001]. The ratio did not change because by 10 min in DA, average peak LP *I*_h_ was significantly and stably reduced to 87 ± 3% of its original value [**Figure 5D**, repeated measures ANOVA, *F*(3,4) = 6.983, *p* = 0.0057]. We conclude that the AD mechanism that regulates LP *I*_h_ *G*_max_ in the presence of DA integrates information on both neuronal burst duration and cycle period.

### SPIKE ACTIVITY DELAYS THE EFFECT OF CHANGES IN SLOW WAVE ACTIVITY

Overall, the data supported our hypothesis: in the presence of 5 μM DA and average DA-induced changes in LP slow wave activity, the DA-induced fold-change in LP *I*_A_ was compensated by a similar fold-change in LP *I*_h_. However, one aspect of the data did not fit with our working model. The ratio could be restored by 10 min (**Figure 5**), but phase recovery required 60 min on average (Figures 1B,D). It is possible that restoration of the LP *I*_A_:*I*_h_ ratio was necessary (**Figure 4**) but not sufficient for phase recovery, and that one or more unidentified slower processes were also involved. Alternatively, one major difference between the experiments shown in Figures 1 vs. 5 was the presence vs. absence of spike activity. If a Ca^2^^+^ sensor participated in this homeostatic mechanism to maintain the LP *I*_A_:*I*_h_ ratio (Gunay and Prinz, 2010), then spike activity and DA-induced changes in slow wave activity might have opposing effects on steady-state Ca^2^^+^, and spike activity could delay the compensatory decrease in LP *I*_h_ by slowing the rate of change of steady-state Ca^2^^+^. To investigate this idea, we repeated experiments to measure the LP *I*_A_:*I*_h_ ratio using a recurring step that mimicked not only slow wave activity, but also, spike activity.

During normal LP activity, spikes passively spread to the soma and neuropil from a distal spike initiation zone (siz). We mimicked spike activity generated at the siz with depolarizing current injections into the soma. We reasoned that LP HCN channels, which are located in the neuropil (Goeritz et al., 2011), will experience a similar depolarization regardless of whether the spikes initiate at the soma or siz, because the two structures are roughly equidistant from the neuropil. This logic rests on the untested assumption that the electrotonic properties and protein composition of the entire primary neurite membrane between soma and spike initiation zone are homogeneous and that electrotonic potentials spread with similar efficiency in both directions. We also made untested assumptions about LP spike amplitude and duration. Peak voltage (+40 mV) and duration (2 ms) of PD spikes have been directly measured from intra-axonal recordings (Ballo et al., 2012). We assumed LP and PD spikes would be similar and used these values here.

Previous work suggested that activity-dependent regulation can be coded by the pattern of spike activity and not simply the total amount of depolarization (Gorbunova and Spitzer, 2002). We performed two series of experiments to determine if spike activity influenced the LP *I*_A_:*I*_h_ ratio either by the total amount of depolarization produced or by the pattern of depolarization. The total amount of depolarization was mimicked with a step to +40 mV whose duration equaled the average number of spikes per burst multiplied by 2 ms. Patterned spike activity was mimicked by 2 ms depolarizations to +40 mV separated by the average interspike interval (ISI), and the number of depolarizations was equal to the average number of spikes per burst.

**FIGURE 6:**
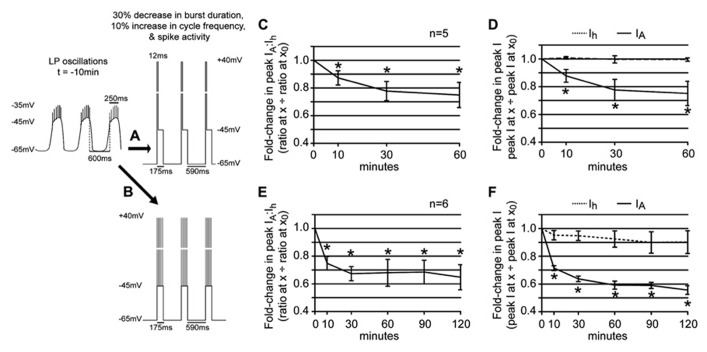
**Spike activity influences the LP *I*_A_:*I*_h_ ratio in 5 μM DA. (A,B)** Diagrams of recurrent voltage steps that were applied during 5 μM DA application. Spikes are not drawn to scale. Note the recurrent step mimicked the DA-induced decrease in LP burst duration and cycle period. In addition, it mimicked spike activity. In **(A)**, spike activity is represented as a single depolarizing step to +40 mV. The duration of the step = 6 spikes × 2 ms = 12 ms. In **(B)**, the six spikes are represented as 6, 2 ms depolarizations to +40 mV. The time between each depolarization is 0.66 *x* average ISI in ms at *t* = -10 min. **(C)** Plot of the fold-changes in the LP *I*_A_:*I*_h _ratio (mean ± SEM) throughout a 1 h superfusion with 5 μM DA and implementation of the recurrent voltage step indicated by protocol A. The ratio significantly decreased with time; *p < 0.05, repeated measures ANOVA with Dunnett’s *post hoc* tests that compare all time points to *t* = 0 (see text). **(D)** Plots of the fold-changes in peak LP *I*_A_ and *I*_h_ (mean ± SEM) from the same experiments as in **(C)**; *p < 0.05 for *I*_A_ only, repeated measures ANOVAs with Dunnett’s *post hoc* tests (see text). **(E)** Plot of the fold-changes in the LP *I*_A_:*I*_h _ratio (mean ± SEM) throughout a 2 h superfusion with 5 μM DA and implementation of a recurring voltage step indicated by protocol B. The ratio significantly decreased with time; *p < 0.05, repeated measures ANOVA with Dunnett’s *post hoc* tests that compare all time points to *t* = 0, *F*(5,4) = 8.728, *p* = 0.0002. **(F)** Plots of the fold-changes in peak LP *I*_A_ and *I*_h_ (mean ± SEM) from the same experiments as in **(E)**. Note that although LP *I*_h_ is slowly reduced, repeated measures ANOVAs with Dunnett’s *post hoc* tests that compare all time points to *t* = 0 indicate that only the decrease in LP *I*_A_ is statistically significant [LP *I*_A_: *F*(3,4) = 19.66, *p* < 0.0001; LP *I*_h_, *F*(3,4) = 1.218, *p* = 0.3456]; *p < 0.05.

In the first set of experiments a depolarizing step to +40 mV was superimposed upon the recurrent voltage step that mimicked LP slow wave activity in 5 μM DA (**Figure 6A**). The duration of the step to +40 mV corresponded to the average number of spikes per burst at *t* = -10 min multiplied by 2 ms. Note that the average number of spikes per burst does not change significantly during a 1 h 5 μM DA application [repeated measures ANOVA, *F*(6,8) = 0.8920, *p* = 0.5083, data not shown]. Surprisingly, this short depolarization on top of the usual recurrent voltage step that mimicked a 30% decrease in LP burst duration and a 10% increase in cycle frequency completely abolished the effect of DA-induced changes in slow wave activity upon LP peak *I*_h_. The LP *I*_A_:*I*_h_ ratio significantly decreased under these conditions [**Figure 6C**; repeated measures ANOVA: *F*(3,3) = 6.114, *p* = 0.0149] because, there was no reduction in LP *I*_h_ (**Figure 6D**, mean ± SEM fold-change in LP peak *I*_h_ at 10 min = 1.008 ± 0.010). The insignificant change in LP *I*_h_ throughout the 1 h 5 μM DA application could not compensate for the significant decrease in LP *I*_A_ [**Figure 6D**; repeated measures ANOVAs: *I*_h_, *F*(3,4) = 0.1801, *p* = 0.9078; *I*_A_, *F*(3,3) = 5.251, *p* = 0.0228]. Note that the change in LP *I*_A_ was not significantly different between experiments that did (**Figure 6D**) vs. did not (**Figure 5D**) mimic spike activity along with DA-induced changes in slow wave activity [two-way ANOVA: treatment, *F*(1,28) = 0.08, *p* = 0.7789; time, *F*(3,28) = 6.83, *p* = 0.0014; interaction, *F*(3,28) = 0.33, *p* = 0.8065].

We next asked if we could delay, but not abolish the compensatory decrease in LP *I*_h_ *G*_max_ by better mimicking the spike pattern (**Figure 6B**). To do this, we included an ISI in between each 2 ms depolarization to +40 mV that was equal to the average ISI at *t* = -10 min multiplied by 0.66, because a 1 h 5 μM DA application reduced the mean ISI to 66% of its initial value [repeated measures ANOVA: *F*(6,4) = 4.002, *p* = 0.0065, data not shown]. Including patterned spike activity in the recurrent voltage step delayed the compensatory reduction in LP *I*_h_ *G*_max_ (**Figure 6F**). By 10 min in 5 μM DA, the compensatory reduction in LP peak *I*_h_ was significantly smaller for protocols that did (**Figure 6F**) vs. did not (**Figure 5D**) include patterned spike activity on top of the slow wave (Student’s *t*-test, *p* = 0.0014). Although a delayed and slowly developing compensatory reduction in LP *I*_h_ *G*_max_ was elicited with protocol B, it was not large enough to compensate for the decrease in LP *I*_A_, even by 2 h (**Figure 6E**). This is because the patterned spike activity also unexpectedly regulated LP *I*_A_: the reduction in peak LP *I*_A_ was significantly larger for protocols that did (**Figure 6F**) vs. did not (**Figure 5D**) include patterned spike activity on top of the slow wave [two-way ANOVA, treatment, *F*(1,32) = 25.76, *p* < 0.0001; time, *F*(3,32) = 38.53, *p* < 0.0001; interaction, *F*(3,32) = 3.45, *p* = 0.0278]. This large decrease in LP *I*_A_ was most likely a technical artifact. Kv4 channels are located throughout the LP somatodendritic membrane (Baro et al., 2000). According to our untested assumption, Kv4 channels in the neuropil will experience typical changes in membrane potential with each spike mimic; however, this is not true for somatic Kv4 channels. The average LP membrane potential typically recorded at the soma at the peak of spike activity is -36 ± 4 mV because spikes are severely attenuated as they passively spread to the soma. Thus, a +40 mV depolarization at the soma is unrealistic and most likely generates an artificially large decrease in the somatic LP *I*_A_. Nonetheless, based on these experiments we can conclude that DAD regulation of LP *I*_h_ *G*_max_ integrates information on burst duration, cycle period, and spike activity.

## DISCUSSION

**FIGURE 7:**
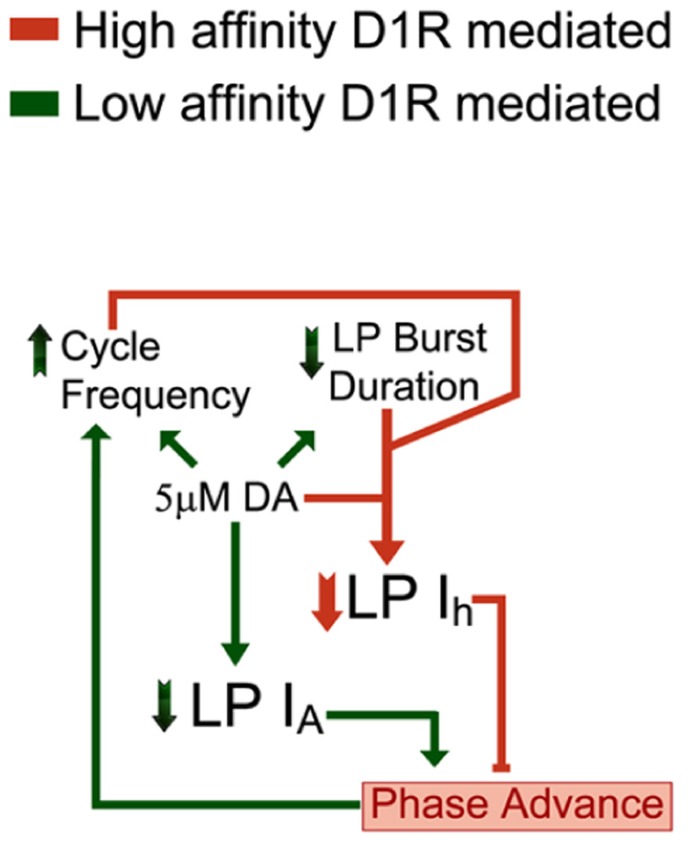
**DA (5 μM) activates a closed loop.** DA (5 μM) acts at high affinity D1Rs to confer activity-dependence upon LP *I*_h_ (DAD regulation, coral). In addition, 5 μM DA acts at low affinity D1Rs to modulate LP *I*_A_ and circuit output (DA modulation, green). Note that the D1R high affinity (coral) and low affinity (green) effects each provide an arm of a closed loop. DA (5 μM) initially increases network cycle frequency, decreases LP burst duration and advances LP activity phase. The latter is due to a decrease in LP *I*_A_. The phase advance not only prevents LP network function, which is to act as a brake on increasing cycle frequencies, but may even drive further increases in cycle frequency. DAD regulation permits these DA-induced changes in activity to subsequently produce a compensatory decrease in LP *I*_h_ *G*_max_. This restores the LP *I*_A_:*I*_h_ conductance ratio and the timing of LP activity phase at the increased cycle frequency and decreased burst duration. This will stabilize circuit output by limiting further increases in cycle frequency.

The principal finding of our study is that 5 μM DA simultaneously creates flexibility and stability in a rhythmically active neural network by activating a closed loop (**Figure 7**). DA acts at both low and high affinity D1Rs to alter activity and enable AD intrinsic plasticity, respectively. The feedback loop re-established a conductance ratio that was modified by DA, and thereby restored a neuronal phase relationship during a sustained increase in cycle frequency. The generation of closed loops via modulator-enabled AD intrinsic plasticity may represent a fundamental organizing principle used by modulatory systems to preserve conductance ratios and their associated activity correlates, while at the same time altering other aspects of circuit output.

### DA SIMULTANEOUSLY GENERATES FLEXIBILITY AND STABILITY BY ACTIVATING HIGH AND LOW AFFINITY D1Rs

Like most systems, DA transmission takes two forms in the stomatogastric nervous system, tonic, and phasic. DA neurons in the commissural ganglia project to the STG and release DA into open synapses; DA then diffuses to its sites of action before re-uptake (Oginsky et al., 2010). To the best of our knowledge, DA levels have not been measured in the STG, but in other systems that use volume transmission, DA is tonically present at ~nM levels (range: 0.1–100 nM) and can transiently increase to ~μM levels (range: 0.1–100 μM) near the release sites of bursting DA neurons (Zoli et al., 1998; Schultz, 2007; Fuxe et al., 2010). In addition, the STG is located in a blood vessel and is bathed by neurohormonal DA (Sullivan et al., 1977; Marder and Bucher, 2007). Generally speaking, high affinity receptors respond to ~nM DA (tonic) and low affinity receptors respond to ~μM DA (phasic). We have previously shown that LP possesses both high and low affinity D1Rs that mediate different effects on *I*_A_. High affinity receptors were activated by a tonic 1 h application of 0.5 nM but not 0.05 nM DA and produced a persistent (i.e., non-reversible) increase in LP *I*_A_ through a translation-dependent mechanism (Rodgers et al., 2011b,in press). On the other hand, low affinity D1Rs responded to bath application of ~μM DA and immediately and reversibly decreased LP *I*_A_ by altering its biophysical properties (Zhang et al., 2010). In this study we showed that high affinity D1Rs do not simply act through slow mechanisms (hours) to produce persistent changes in ionic currents, but can also rapidly (seconds to minutes) confer activity-dependence upon an ionic conductance to generate a feedback loop.

Concomitant stimulation of both low and high affinity LP D1Rs activates a closed loop that maintains neuronal activity phase while other aspects of neuronal output are altered (**Figure 7**). A 5 μM but not 5 nM DA application alters pyloric network activity (Rodgers et al., 2011a); therefore, DA acts at low affinity receptors to modulate circuit output. At least three key aspects of pyloric network output are modulated by DA (Rodgers et al., 2011a): on average, cycle frequency is increased by ~10%, LP burst duration is decreased by 30% and LP firing phase is advanced by ~20%. The LP phase advance is largely due to a DA-induced reduction in LP *I*_A_ (Harris-Warrick et al., 1995; Zhang et al., 2010). These alterations in network output disrupt LP network function (Johnson et al., 2011). Normally, LP acts through the LP–PD synapse to slow increasing cycle frequencies (Nadim et al., 1999; Weaver and Hooper, 2003; Mamiya and Nadim, 2004, 2005; Johnson et al., 2011). The timing of LP activity phase is critical for this function because, LP inhibition has different effects according to when it occurs during the pacemaker oscillation, and a phase advance can even increase cycle frequency (Thirumalai et al., 2006; Johnson et al., 2011). This creates a potential for spiraling changes in network output that would destabilize the system. However, besides eliciting these alterations in network activity, DA acts at high affinity D1Rs to permit AD regulation of LP *I*_h_. This allows the DA-induced changes in cycle frequency and LP burst duration to subsequently elicit a reduction in LP *I*_h_ that exactly compensates for the modulatory decrease in LP *I*_A_ to restore the timing of LP activity phase. Restoring LP firing phase re-establishes LP network function which is to slow increasing cycle frequency (Johnson et al., 2011). This could limit the DA-induced increase in cycle frequency driven by DA actions on the pacemaker and stabilize circuit performance at the increased network cycle frequency, decreased LP burst duration, and potentially altered LP input:output gain (Burdakov, 2005). It should also restore the initial phasing of rhythmic pyloric muscle contractions, but at an increased cycle frequency. Interestingly, burst duration and on/off-delays scale with cycle period in the natural population throughout development and over a wide range of temperatures (Bucher et al., 2005; Goaillard et al., 2009; Tang et al., 2010). Thus, the closed loop uncovered here may be part of a more extensive control system that synchronizes these network characteristics over multiple time scales and through multiple mechanisms.

### DOPAMINERGIC TONE MIGHT MAINTAIN THE *I_A_*:I_h_ RATIO DURING NON-DOPAMINERGIC PERTURBATIONS TO ACTIVITY

Landmark studies from the Marder group demonstrated equivalent neuronal and network firing patterns can arise from different sets of intrinsic and synaptic conductances (Golowasch et al., 1999a,b; Prinz et al., 2004b; Schulz et al., 2006, 2007). This work led to the idea that conductances co-vary over time in order to maintain a particular activity feature, an idea that was supported by existing ion channel overexpression studies (MacLean et al., 2003, 2005). These findings were unexpected and caused the Selverston group to ask: can the output of a network made up of disparate components be robust to perturbation (Szucs and Selverston, 2006)? Within a population, peak PD *I*_A_ and PD *I*_h_ each varies by >3-fold across individuals; but, all individuals maintain the same PD *I*_A_:*I*_h_ ratio (Temporal et al., 2012). Selverston’s group reasoned that if PD *I*_A_ were blocked with 4-AP in multiple preparations, then PD *I*_h_ would no longer be balanced in any preparation, and the variable amounts of PD *I*_h_ in each preparation would be revealed in distinct PD activity patterns (Szucs and Selverston, 2006; Nowotny et al., 2007). To their surprise, all blocked neurons produced similar activity patterns, suggesting either that the pyloric network is not made up of disparate components in each individual or that rapid compensatory mechanisms must exist to maintain activity. Our findings suggest the latter may be true: modulator-enabled, AD feedback loops could have produced compensatory changes in *I*_h_ *G*_max_ that maintained activity in the Selverston group’s experiments. Indeed, modulatory inputs were intact in the latter studies (Szucs and Selverston, 2006), and 4-AP significantly alters pyloric cycle period and neuronal burst durations (Tierney and Harris-Warrick, 1992). Together, the data imply that modulatory tone could enable multiple AD feedback loops that maintain conductance ratios and their activity correlates under a variety of conditions.

### PHASE MAINTENANCE

Both intrinsic and synaptic mechanisms can operate over different time scales to maintain pyloric neuron phase relationships when cycle frequency varies. Synaptic depression rapidly promotes phase maintenance by proportionately delaying neuronal firing as synapses increasingly recover from depression with longer cycle periods (Nadim et al., 1999, 2003; Manor et al., 2003). DA can modulate synaptic dynamics to promote phase maintenance: 10 μM DA decreased the time constants of short-term depression and its recovery at the PD–LP graded synapse, thus contributing to phase maintenance with changing network frequency (Kvarta et al., 2012). It is also worth noting that PY inhibition onto LP plays an important role in determining LP off-phase and this impact is enhanced in DA (Johnson et al., 1993, 1995), contributing to the shortening and stabilization of LP activity phase (Johnson et al., 2011). Fast intrinsic conductances, including *I*_A_, can act in conjunction with synaptic mechanisms to promote phase maintenance in pyloric neurons (Bose et al., 2004; Greenberg and Manor, 2005; Rabbah and Nadim, 2005). Slower processes can also play a role in pyloric neuron phase maintenance. In a combined physiological and computational study on the spiny lobster, Hooper et al. (2009) demonstrated that a conductance with slow activating and inactivating kinetics (seconds to minutes) could explain adjustment of PIR and phase maintenance in PY neurons in the presence of altered cycle period. Goaillard et al. (2010) showed the crab LP neuron possessed a similar mechanism. Neither of these studies identified the slow conductance. *I*_h_ was considered, but blocking *I*_h_ did not terminate the mechanism. The authors suggested the conductance could be an unidentified slow potassium or calcium conductance, deinactivation of a fast sodium current, a pump current or a combination of opposing currents with fast kinetics. Our research extends these previous findings by revealing the existence of a DA-enabled mechanism(s) for phase maintenance that involves preserving the *I*_A_:*I*_h_ ratio. DAD regulation of *I*_h_ may contribute to phase maintenance in other rhythmically active systems where phase relationships are maintained amidst changes in cycle frequency (Dicaprio et al., 1997; Jacobson et al., 2009).

### MECHANISM OF DAD REGULATION OF LP *I_h_*

DAD regulation of LP *I*_h_ *G*_max_ integrates information on multiple aspects of activity. The neurons under study exhibit slow membrane potential oscillations (~20 mV at 1–2 Hz) and action potentials riding on the depolarized plateau of each oscillation. DAD regulation integrated information on cycle period and burst duration, as well as spike activity. Integration may be an epi-phenomenon created by voltage clamp measures of the entire population of HCN channels, and it is possible that distinct subcellular populations of HCN channels are differentially regulated by different types of activity.

It is not clear if DAD regulation of LP *I*_h_ represents a single integrator that is influenced by multiple types of activity; or, if multiple molecular integrators exist, each of which is sensitive to a distinct aspect of activity. AD mechanisms that regulate *I*_h_ density could rely on both Ca^2^^+^ release and Ca^2^^+^ entry. It is tempting to speculate that the mechanism(s) that is sensitive to burst duration and cycle frequency senses Ca^2^^+^ release from stores while the mechanism(s) that is sensitive to spiking senses Ca^2^^+^ entry through voltage-gated calcium channels. It was previously noted that Ca^2^^+^ release from stores can regulate *I*_h_ density in hippocampal neurons (Narayanan et al., 2010), and that in the pyloric AB neuron, Ca^2^^+^ release oscillates with oscillations in membrane potential (Kadiri et al., 2011). Thus, changes in cycle frequency and burst duration could alter steady-state Ca^2^^+^ contributed by store release. In addition, Ca^2^^+^ entry through glutamate receptors can regulate surface expression of HCN channels over minutes in cultured hippocampal neurons (Noam et al., 2010). Perhaps this mechanism may be generalized to Ca^2^^+^ entry through other types of channels, such as high threshold voltage-gated Ca^2^^+^ channels that open maximally during spike activity. In this case, spike frequency could also influence steady-state Ca^2^^+^. Previous studies show that micromolar DA can enhance LP voltage-gated Ca^2^^+^ currents (Johnson et al., 2003; Kloppenburg et al., 2007), and in the AB neuron micromolar DA can act on IP_3_ receptors to increase release from stores (Kadiri et al., 2011). Since higher concentrations of DA can alter Ca^2^^+^ dynamics, these data suggest that DAD regulation of LP *I*_h_ may vary according to DA concentrations as well as activity patterns.

The mechanisms by which high affinity D1Rs permit AD regulation of LP *I*_h_ *G*_max_ are not known. Traditionally, D1Rs are thought to act through Gαs to regulate adenylyl cyclase activity and thereby cAMP levels, which in turn regulate PKA. D1R induced increases in PKA activity can regulate surface expression of cortical neuron glutamate receptors (Sun et al., 2005). Thus, in one scenario, a cAMP-PKA signaling pathway may modulate AD surface expression of HCN channels. Indeed such a pathway can regulate AD Kv4 channel trafficking in hippocampal neurons (Hammond et al., 2008). One of the invertebrate adenylyl cyclases, rutabaga, is a known coincidence detector that can be influenced by both Gαs and Ca^2^^+^ (Tomchik and Davis, 2009; Gervasi et al., 2010), and rutabaga could underpin DA’s permissive effect.

## Conflict of Interest Statement

The authors declare that the research was conducted in the absence of any commercial or financial relationships that could be construed as a potential conflict of interest.
